# Effects of Performance Variations in Key Components of CRTS I Slab Ballastless Track on Structural Response Following Slab-Replacement Operations

**DOI:** 10.3390/ma18153621

**Published:** 2025-08-01

**Authors:** Wentao Wu, Hongyao Lu, Yuelei He, Haitao Xia

**Affiliations:** 1 College of Urban Rail Transportation, Shanghai University of Engineering Science, Shanghai 201600, China; wu.wentao@sues.edu.cn (W.W.); hyldoc@163.com (Y.H.); 2 Shanghai Key Laboratory of Structural Durability and System Safety of Rail Transit, Tongji University, Shanghai 201800, China; 3 China Railway Shanghai Group Co., Ltd., Shanghai 201800, China; 13761313651@163.com

**Keywords:** ballastless track slab, slab-replacement operation, key components, quantification of variations, path of force transmission

## Abstract

Slab-replacement operations are crucial for restoring deteriorated CRTS I slab ballastless tracks to operational standards. This study investigates the structural implications of the operation by evaluating the strength characteristics and material properties of track components both prior to and following replacement. Apparent strength was measured using rebound hammer tests on three categories of slabs: retained, deteriorated, and newly installed track slabs. In addition, samples of old and new filling resins were collected and tested to determine their elastic moduli. These empirical data were subsequently used to develop a refined finite-element model that captures both pre- and post-replacement conditions. Under varying temperature loads, disparities in component performance were found to significantly affect stress distribution. Specifically, before replacement, deteriorated track slabs exhibited 10.74% lower strength compared to adjacent retained slabs, whereas, after replacement, new slabs showed a 25.26% increase in strength over retained ones. The elastic modulus of old filling resin was measured at 5.19 kN/mm, 35.13% below the minimum design requirement, while the new resin reached 10.48 kN/mm, exceeding the minimum by 31.00%. Although the slab-replacement operation enhances safety by addressing structural deficiencies, it may also create new weak points in adjacent areas, where insufficient stiffness results in stress concentrations and potential damage. This study offers critical insights for optimizing maintenance strategies and improving the long-term performance of ballastless track systems.

## 1. Introduction

CRTS I slab ballastless track has been widely adopted in high-speed railway construction owing to its high stability, durability, and low maintenance requirements. In this system, track slabs are individual units interconnected by convex sets limit devices, with filling resin serving as a cushion material at the interface between the limit devices and the track slab. During long-term service, the coupled action of train dynamic loads and temperature loads induces alterations in the microstructure of internal materials, which in turn compromise the integrity of critical components or localized regions, potentially leading to damage or failure [1,2]. To ensure that CRTS I slab ballastless track structures continue to meet service requirements and to prevent the deterioration of load-bearing capacity and geometric alignment, the Chinese railway authorities have implemented slab-replacement operations for panels exhibiting cracks, voids, or map-like spalling.

Extensive research has been conducted on damage detection, service performance evaluation, and slab-replacement procedures for railway track structures. In the field of damage detection for track slabs [3,4,5], scholars have applied a variety of advanced techniques, including image recognition [6,7,8], 3D laser scanning [9,10], and ultrasonic testing [11,12,13,14], to carry out comprehensive assessments of track slab defects under in-service conditions. These studies have successfully identified the characteristic features of damage [15,16,17,18] and the laws of crack propagation [19,20,21], thereby achieving both quantitative evaluations of surface deterioration and visualizations of internal defects in ballastless track slabs. Based on the outcomes of these damage detection efforts, researchers have developed deterioration classification systems by integrating surface-level indicators [22,23,24] and internal performance metrics [25,26,27]; they have also proposed predictive models to estimate the remaining service life of the slabs [28,29]. When deteriorated track slabs continue to operate after undergoing maintenance, their performance tends to degrade progressively under the combined influence of multiple environmental and mechanical factors. In engineering practice, the Chinese railway authorities primarily evaluate the residual load-bearing capacity of the slabs by assessing the number and severity of surface cracks and spalling [30,31,32,33], based on which they classify slab conditions and formulate rational replacement strategies [34,35,36,37]. On this basis, researchers have primarily focused on the performance assessment and evaluation of ballastless track slabs in service. However, limited attention has been given to the service performance of CRTS I slab-type ballastless track structures after continued degradation, especially concerning the mismatches in material properties and mechanical strength between new and old components introduced by slab-replacement operations.

**Figure 1:**
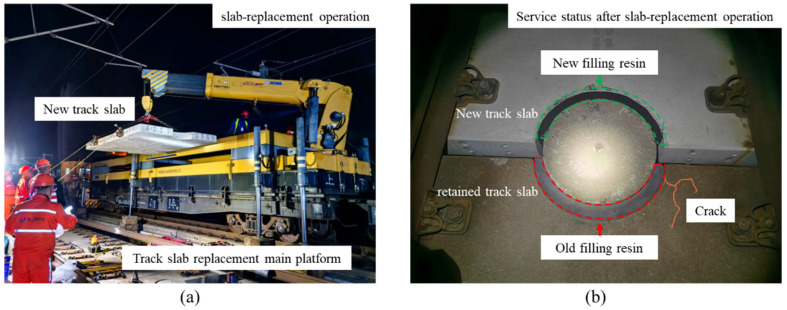
Slab-replacement operation of CRTS I slab ballastless track: (**a**) diagram of slab-replacement operation; (**b**) structural differences and damage after slab-replacement operations.

During slab-replacement operations, the old filling resin adjacent to deteriorated track slabs must be removed via chiseling to prevent excessive interlayer compression caused by ambient temperature fluctuations during exposure [38,39,40]. After the deteriorated track slab is extracted and the new track slab is positioned and fine-aligned, fresh filling resin is cast in place, resulting in structural strength and resin-modulus disparities across the limit devices. Consequently, the longitudinal arrangement of ballastless track slabs shifts from a “retained track slab–deteriorated track slab–retained track slab” configuration to a “retained track slab–new track slab–retained track slab” connection pattern. It is noteworthy that the retained track slabs flanking the replaced slab have endured years of loading and environmental exposure, leading to reductions in concrete strength and resin stiffness relative to the newly installed slab. Such mismatches produce abrupt changes in longitudinal track stiffness and interfacial constraint conditions, directly altering load-transfer paths and potentially precipitating secondary defects. As shown in Figure 1b, field observations have recorded cracking in the retained track slabs adjacent to the new panel near the limit devices. These findings highlight the need for a systematic investigation of post-replacement performance disparities in CRTS I slab ballastless track components to clarify their effects on structural force transmission.

To address this need, rebound-hammer tests were performed on retained, deteriorated, and new track slabs within the replacement zone, and laboratory measurements of the elastic moduli of both old and new filling resins were carried out to quantify key component performance parameters before and after replacement. These quantified data were then incorporated into a refined finite-element model reflecting both aged and new component conditions, and stress distributions under varying thermal loads were analyzed. The results elucidate how the material and structural discontinuities introduced by slab-replacement operations influence secondary damage mechanisms in ballastless tracks and provide a theoretical basis for guiding targeted inspections of weak points following slab-replacement operations.

## 2. Quantitative Tests on the Differences in Key Components Before and After Slab-Replacement Operations

Track slabs serve as the primary load-bearing elements in ballastless track structures, supporting train loads and transmitting them to the underlying foundation. Filling resin functions as the critical restraint and cushioning component, effectively mitigating excessive lateral and longitudinal displacements of track slabs under the combined action of thermal and dynamic loads, which would otherwise impose over-compression on limit devices’ side faces and shear stresses at their bases. As key constituents of the CRTS I slab ballastless track, track slabs and filling resin play essential roles in maintaining structural stability, attenuating train-induced vibrations, and prolonging track service life. Consequently, it is necessary to quantify both the apparent strength variations in slabs and the elastic modulus differences in filling resins within the slab-replacement zone.

### 2.1. Quantification Method for Overall Structural Strength Differences

**Figure 2:**
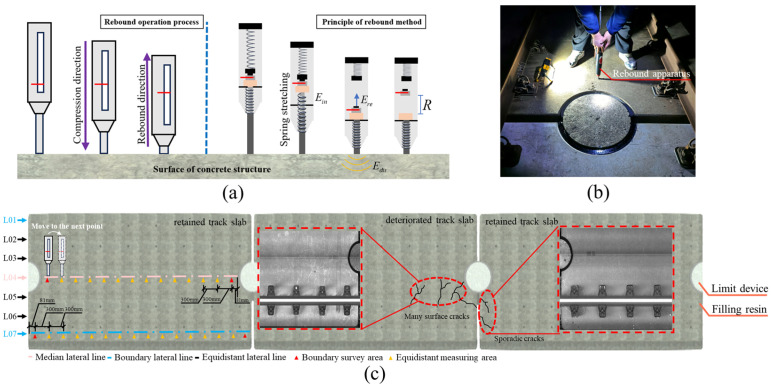
Apparent strength characterization test of the track slab: (**a**) the apparent strength is detected by the rebound tester; (**b**) schematic diagram of the rebound method; (**c**) layout of the apparent strength measurement of the track slab.

As illustrated in Figure 2a, the rebound method is a nondestructive test that exploits the correlation between surface hardness and mechanical properties. A spring-driven hammer delivers a constant initial kinetic energy impact to the concrete surface; some energy is dissipated through plastic deformation and acoustic emission, and the remaining energy causes the hammer to rebound. Because rebound height is closely linked to the material elastic modulus, compressive strength, and microstructural characteristics, a quantitative empirical model relating rebound number ($R_{m}$) to concrete compressive strength ($f_{cu}^{i}$) may be established via calibration curves. In accordance with the “Technical Specification for Strength Testing of Concrete Components by Rebound Method” [41], rebound measurements were conducted on the deteriorated track slabs, the adjacent retained track slabs, and the new track slabs awaiting service, with the field layout depicted in Figure 2b. To avoid rebounding over internal reinforcement, test lines were arranged longitudinally along each slab. Seven transverse test lines 01~07 were evenly spaced at 300 mm intervals: lines 01 and 07 were boundary lines located 150 mm from the slab edges, and line 04 was the centerline. Along each test line, equidistant measuring areas were positioned at 300 mm spacing; the boundary survey area was set 81 mm from the slab ends to clear the limit devices. The full grid of the test locations is shown in Figure 2c. A total of six deteriorated slabs, six newly replaced slabs, and twelve adjacent in-service slabs (six on each side) were tested.

### 2.2. Quantification Method for the Difference in Elastic Coefficient of Filled Resin

**Figure 3:**
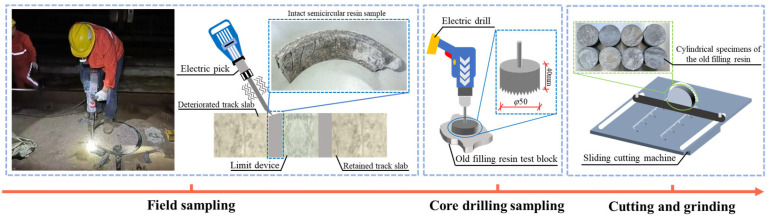
Sampling process of old filling resin cylindrical test blocks.

The preparation process for cylindrical specimens of the old filling resin is illustrated in Figure 3. Prior to the slab-replacement operation, the old filling resin adjacent to the track slabs slated for replacement was chiseled off. Intact semicircular resin samples were retrieved from the construction site. Due to the curvature and concave surface of these semicircular specimens, they could not be processed into standard cubic specimens in accordance with the “Provisional Technical Specifications for Polyurethane Resin for Convex Stops” [42]. Consequently, a core drill with a diameter of φ 50 mm was employed to extract cylindrical cores from the old filling resin. During the drilling process, the core-drilling machine was securely positioned on the surface of the resin samples. To ensure uniform height-to-diameter ratios among the specimens, a sliding saw was used to trim the cores to the desired height. The final cylindrical specimens of the old filling resin measured 50 mm in diameter and 25 mm in height. Prior to conducting the elastic modulus tests, the specimen surfaces were cleaned thoroughly, and their top and bottom faces were ground flat and smooth using a precision grinding machine to ensure surface evenness and quality.

**Figure 4:**
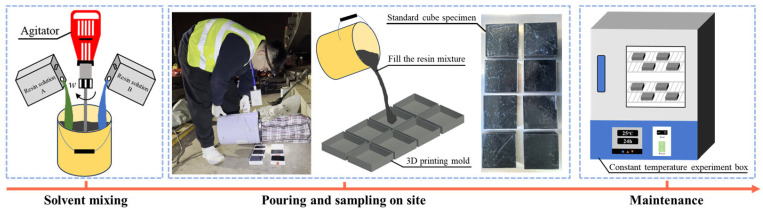
Preparation process of new filling resin standard cubic test blocks.

The casting procedure for standard cubic specimens of the new filling resin is depicted in Figure 4. The resin was prepared by combining solutions A and B in a 1:20 weight ratio. In accordance with the “Provisional Technical Specifications for Polyurethane Resin for Convex Stops” [42], a casting mold with cavity dimensions of 100 mm × 100 mm × 25 mm was fabricated via 3D printing. The mixed resin solution was poured into the mold on-site and allowed to cure until fully solidified. The specimens were then demolded and conditioned for 28 days in an environment maintained at 23 °C ± 2 °C and 50% ± 10% relative humidity.

**Figure 5:**
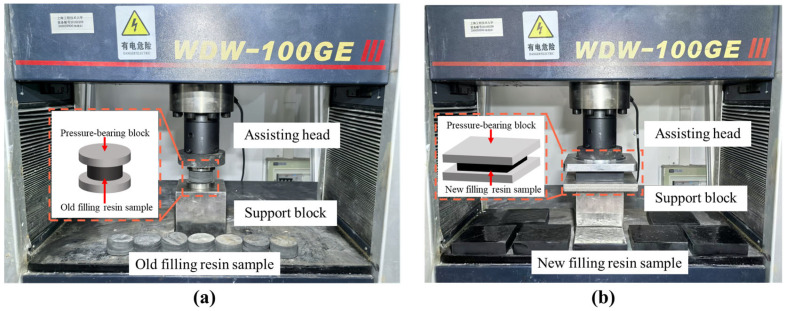
Experiment on the elastic coefficient of new and old filling resins: (**a**) experimental setup for elastic coefficient of cylindrical filling resins; (**b**) experimental setup for the elastic coefficient of standard cubic filling resin.

As shown in Figure 5, elastic modulus tests were conducted on filling resin specimens, with eight new and eight old samples placed sequentially on the support blocks of a universal testing machine. Prior to each test, specimens were aligned to ensure intimate contact between their top and bottom faces and the machine’s loading head and pressure-bearing block. Specimen geometric parameters and testing settings were then determined: the displacement control mode was selected, and a loading rate of 1 mm/min was prescribed. Each specimen was preloaded twice between 0 kN and 4.5 kN before the formal test. Thereafter, the elastic modulus of both new and old filling resin materials was determined with high accuracy.

## 3. Analysis of Quantification Results of Key Component Differences

In order to quantify post-replacement disparities in critical ballastless track components, rebound-hammer tests were conducted on deteriorated track slabs, new installed track slabs, and adjacent retained track slabs to estimate concrete compressive strength and thereby quantify variations in structural capacity. Elastic modulus tests were likewise performed on new and aged filling-resin specimens to determine their respective elastic coefficient and quantify differences in material elasticity.

### 3.1. Numerical Analysis of Strength Difference of Track Slabs

**Figure 6:**
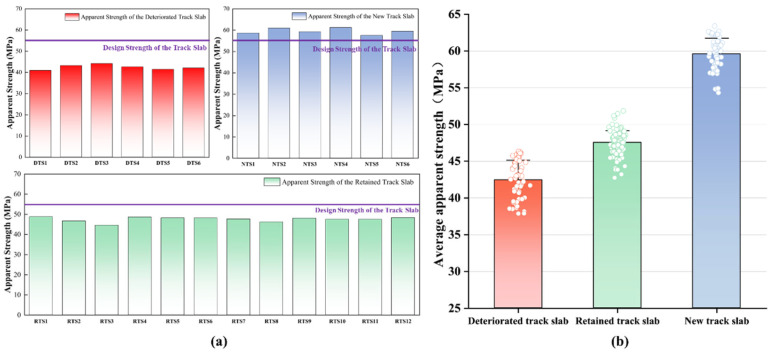
Statistical chart of the apparent strength of the track slab: (**a**) statistics of apparent strength of track slabs in different service states; (**b**) average apparent strength of track slabs in different service states.

According to the “Technical Specification for Strength Testing of High-Performance Concrete” (JGJ/T294-2013) [43], the mean rebound number ($R_{m}$) of each slab was converted into an estimated compressive strength ($f_{cu}^{i}$). The statistical results of the apparent strength for the deteriorated track slab (abbreviated as DTS in the figure), the new track slab (abbreviated as NTS), and the retained track slab (abbreviated as RTS) are shown in Figure 6a. The estimated strength of the deteriorated track slabs was markedly lower than that of the adjacent retained track slabs, whereas the new track slabs installed after the slab-replacement operation exhibited substantially higher strength. Based on the statistical results of the apparent strength of slabs under different service conditions, the average estimated concrete strength was inferred, as shown in Figure 6b. Specifically, the deteriorated track slabs yielded an estimated strength of 42.4 MPa, representing a 22.9% deficit relative to the design value; the adjacent retained track slabs averaged 47.5 MPa, 13.6% below design value; and the new track slabs achieved a mean strength of 59.5 MPa, exceeding the design requirement by 8.2%.

### 3.2. Numerical Analysis of Elastic Difference of Filled Resin

**Figure 7:**
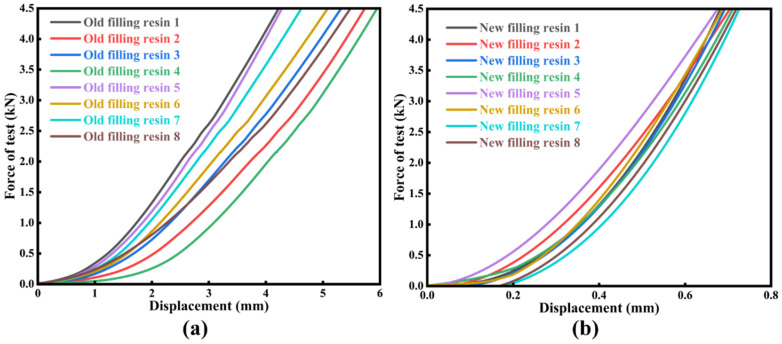
Elastic coefficient experimental curve: (**a**) experimental curve of the old filling resin in the cylinder; (**b**) test curve of new filling resin for standard cubes.

Figure 7 presents the load–deformation curves obtained from elastic-modulus tests on the new and old filling resin specimens. During each formal test, the deformations corresponding to applied loads of 1 kN (P1) and 4 kN (P2) were recorded. Within the proportional limit, the ratio of applied load to the corresponding deformation defines the elastic modulus, E, which was calculated according to Equation (1):

(1) $$k=(P_{1}-P_{2})/\Delta \delta$$

In this equation, Δδ is the specimen deformation between loads P_1_ and P_2_, measured in millimeters.

In this study, the elastic modulus of the old filling resin was quantified using cylindrical specimens, which differ substantially in volume from the cubic specimens prescribed by current standards. To establish an equivalence relationship between specimens of different geometries, conversion criteria for the elastic modulus of nonstandard specimens were derived. Given that both specimen types share the same intrinsic elastic modulus but differ in geometry, analytical elastic–mechanical models were developed for each geometry to derive their modulus expressions, and a conversion equation based on geometric parameters was subsequently formulated.

The stress applied to the upper surface of the filling resin can be calculated based on its definition as follows:

(2) σ=FA

where F is the pressure applied to the upper surface of the filling resin; A represents the contact area of the upper surface of the test block.

Combined with the definition of linear elastic strain, the following relationship is obtained:

(3) ε=Δxh

In this equation, Δx represents the vertical displacement of the test block when it is subjected to vertical pressure; h represents the height of the test block.

According to Hooke’s Law, by combining Equations (2) and (3) and utilizing the definition formula of the elastic modulus E of the filling resin, the conversion equation between the elastic modulus of the filling resin with different geometric characteristics and its elastic coefficient can be derived as follows:

(4) E=khA

where k denotes the elastic coefficient of the test block within the elastic limit.

The elastic modulus of filling resin specimens with different geometries is identical. Thus, the conversion equation between the elastic coefficient of cylindrical specimens and that of standard cubic specimens can be expressed as:

(5) ki=kjhjAihiAj

**Figure 8:**
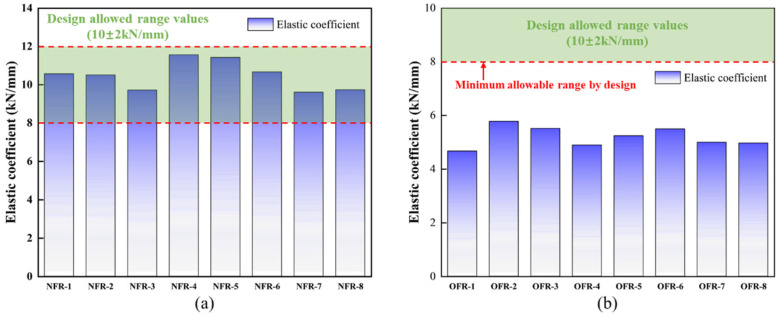
Statistical chart of elastic coefficient of filling resin: (**a**) statistical chart of elastic coefficient of new filling resin; (**b**) statistical chart of the elastic coefficient of old filling resin.

The elastic coefficients obtained from the elastic modulus tests on the aged cylindrical resin specimens were substituted into Equation (5) to calculate the corresponding values for standard cubic specimens. According to the statistical analysis of the elastic modulus of the new filling resin (NFR) and the old filling resin (OFR) as presented in Figure 8, the old filling resin’s stiffness is markedly below the allowable range of 8.00 to 12.00 kN/mm. The minimum value measured for the old filling resin was 4.67 kN/mm, which is 41.63% below the lower design limit, and its mean value was 5.19 kN/mm, 35.13% below that limit. In contrast, the elastic coefficients of the new filling resin conform to the specified range: the maximum reached 11.57 kN/mm, 44.64% above the lower limit, and the mean was 10.48 kN/mm, 31.00% above the lower limit.

### 3.3. Quantitative Results of Track Structure Differences Before and After the Slab-Replacement Operation

**Figure 9:**
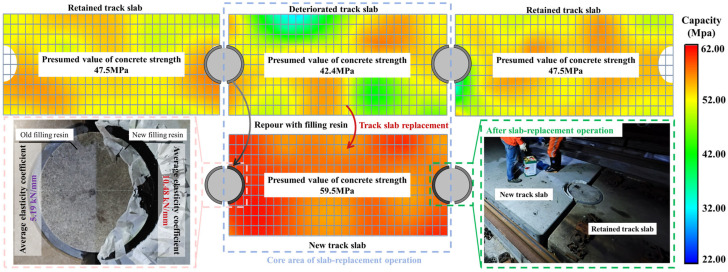
Quantification of the differences in key components of ballastless track before and after the slab-replacement operation.

As shown in Figure 9, the analysis of the quantified track slab apparent strength and filling resin stiffness data reveals pronounced service strength disparities between new and old components adjacent to the limit devices following slab-replacement operations. Prior to replacement, the strength difference between slabs flanking the limit device was 10.74%, whereas, after replacement, this disparity increased to 25.26%. Moreover, the elastic modulus of the old filling resin on one side of the limit device is 101.93% lower than that of the new filling resin on the opposite side. These heterogeneities inevitably induce longitudinal stiffness transitions in the ballastless track, altering its load-bearing behavior and force-transmission paths. On the basis of these pre-replacement and post-replacement component discrepancies, we proceed to analyze the structural response of the ballastless track under varying temperature loads, thereby providing the necessary data support for the finite element simulations presented in the following section.

## 4. Analysis of the Force Characteristics of the Track Slab Before and After the Slab-Replacement Operation

**Figure 10:**
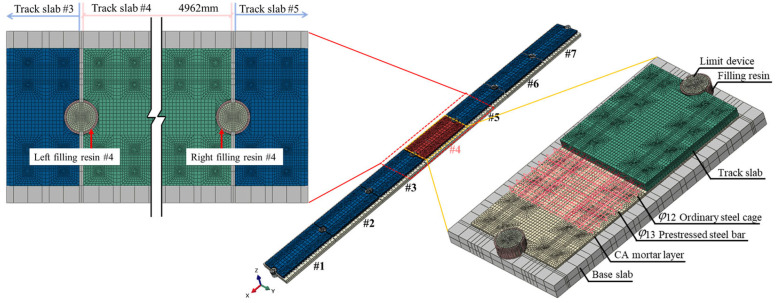
The calculation model of CRTS I slab ballastless track.

To mitigate the influence of boundary conditions, a finite element model comprising seven CRTS I ballastless track slabs was developed using the ABAQUS/CAE 2022 platform, as illustrated in Figure 10. The model includes essential components such as track slabs, mortar layers, base slabs, limit devices, filling resin, prestressed steel bars, and transverse and longitudinal reinforcements. The track slab is a 4962-type prestressed flat slab with dimensions of 4962 mm × 2400 mm × 190 mm, reinforced internally with φ 13 prestressed steel bars and φ 12 conventional steel reinforcements. Both the limit device and base slab are assigned a concrete strength grade of C40. Detailed material parameters are listed in Table 1.

For the mesh discretization, all components except for the reinforcements and steel bars were simulated using eight-node linear hexahedral elements (C3D8R), while the reinforcements and steel bars were represented using two-node linear three-dimensional truss elements (T3D2). To analyze the structural response differences induced by key component discrepancies in CRTS I ballastless track slabs before and after the slab-replacement operation, material parameters were calibrated based on the experimental results presented in Section 3.3.

Specifically, before the slab-replacement operation, the material properties of Track Slab #4 were assigned based on the parameters of the deteriorated slab, while the filling resin on both sides was modeled using the properties of the old filling resin. After the slab-replacement operation, Track Slab #4 was updated with the material parameters of the new track slab, and the adjacent filling resin was modeled using the properties of the new filling resin. For the remaining track slabs and filling resin, the material properties were assigned based on the parameters of the retained track slab and old filling resin, respectively.

This study focuses on the influence of performance differences in key components of ballastless track structures, before and after slab-replacement operation, on the distribution of structural forces through finite element analysis. The primary performance variations introduced by slab replacement occur at the interface between the track slab and the limit device, where both material properties and mechanical strength are altered. These changes result in longitudinal structural discontinuities within the ballastless track system. Accordingly, the finite element simulation emphasizes the analysis of stress variations at the ends of the track slabs. In ballastless track systems, longitudinal forces are predominantly induced by temperature loads, whereas train loads, as high-frequency fatigue actions, mainly contribute to vertical stress responses. Based on this understanding, the present study concentrates on analyzing temperature-induced loading conditions, which are the primary factors affecting the longitudinal mechanical behavior of the structure. To investigate the evolution of stress characteristics in the ballastless track structure under temperature loads after the slab-replacement operation, various temperature load conditions were applied to the track slab model, as specified in the “Code for Design of High-Speed Railways” (TB10621-2014) [44]. The specific loading conditions are detailed in Table 2.

## 5. Analysis of the Stress Distribution Law on the Surface of the Track Slab

This section examines the impact of structural performance differences between new and existing components before and after slab-replacement operations on the surface stress characteristics of the retained track slabs in the CRTS I ballastless track under temperature loading conditions. The slab-replacement operation results in localized reinforcement; however, it also creates new performance disparities and abrupt transitions at the connection interfaces. The retained track slabs on both sides, which remain in service, become the structurally weakest areas, inevitably leading to stress redistribution. This condition deviates from the original design intent of the structure and may result in the emergence of new damage. Fundamentally, this phenomenon arises from the performance disparities at transitional positions, which cause damage to relatively weaker structural components. The analysis emphasizes the stress state and load transfer pathway evolution in the old slabs at the connection interfaces after the installation of new track slabs.

**Figure 11:**
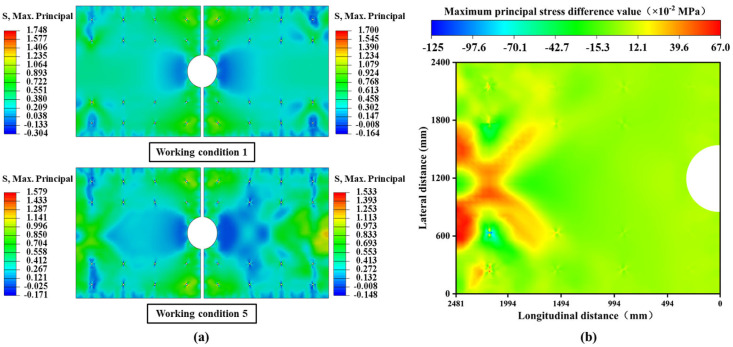
Overall cooling and positive temperature gradient coupling conditions: (**a**) cloud maps of the maximum principal stress distribution in conditions 1 and 5; (**b**) cloud diagram of stress changes in the retained track slab before and after slab-replacement operation.

Under the combined loading conditions of overall cooling and positive temperature gradient, significant asymmetrical differences in stress states are observed both before and after the slab-replacement operation, whether between the “retained track slabs and the deteriorated slabs” or between the “retained track slabs and the new track slabs”. Notably, the replacement of the slab substantially improves the structural stress conditions in terms of magnitude. As illustrated in Figure 11a, taking tensile stress as an example, the maximum tensile stress peak of the new track slab decreases by 0.167 MPa compared to the deteriorated track slab, while the maximum tensile stress peak of the unreplaced, continuously serviced slab decreases by 0.169 MPa. The localized reinforcement effectively reduces the stress levels in the new structure, which is overall beneficial for extending the structural service life.

To further quantify the alterations in the load transfer pathways of the retained track slab, the stress differences at identical node positions before and after the slab-replacement operation were analyzed. As shown in Figure 11b, a significant stress redistribution occurs in the retained track slab. The most notable differences appear in the central regions of the slab. Before the replacement, only two regions exhibited alternating tensile and compressive stress zones. However, after the replacement, four alternating zones emerged. This increase in alternating tensile and compressive stress regions significantly reduces the fatigue limit of the track slabs, potentially leading to premature structural failure before reaching the design service life.

**Figure 12:**
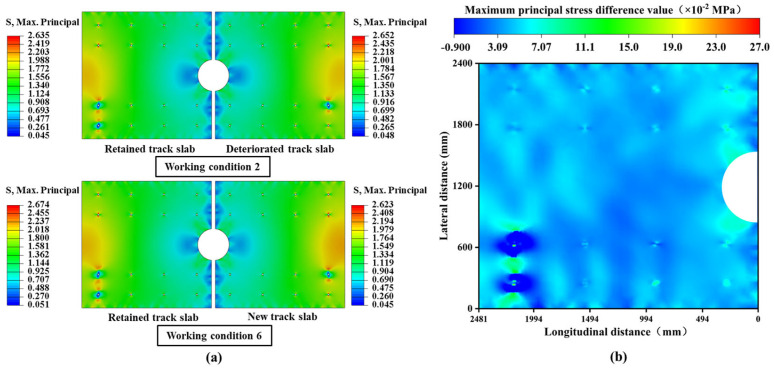
Overall cooling and negative temperature gradient coupling conditions: (**a**) cloud maps of the maximum principal stress distribution in conditions 2 and 6; (**b**) cloud diagram of stress changes of retained track slab before and after slab-replacement operations.

Significantly, under the combined loading conditions of overall cooling and negative temperature gradient, the stress amplitudes are concentrated in structurally weaker areas, as shown in Figure 12a. Before the slab-replacement operation, the maximum stress amplitude is observed in the deteriorated slab, validating the necessity of the slab-replacement operation. Without the slab-replacement operation, the deteriorated track slab would experience accelerated performance degradation under higher stress states, potentially compromising its structural stability. After the replacement, the maximum stress amplitude shifts to the retained track slab, indicating that the slabs adjacent to the newly replaced one have become stress concentration zones. Compared to the pre-replacement condition, the maximum tensile stress on the slabs adjacent to the new slab increases by 0.039 MPa, and the maximum compressive stress rises by 0.006 MPa. This demonstrates that the boundary constraints of the retained track slabs have been altered due to the replacement operation, subjecting these slabs to greater stress amplitudes, which will inevitably reduce their service life.

To further quantify the changes in load transfer pathways for the retained track slab, stress differences at identical node positions before and after slab-replacement operations were analyzed. As shown in Figure 12b, significant stress redistribution occurs in these retained track slabs. Specifically, the tensile stress zones shift toward the end regions near the anchorage structures and bolt sleeves, indicating a heightened risk of tensile cracking at these locations. This damage, being latent in nature, necessitates increased vigilance during subsequent maintenance inspections.

**Figure 13:**
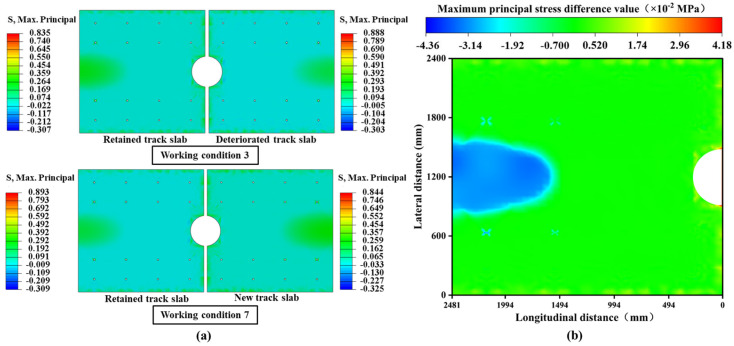
Coupling conditions of overall temperature rise and positive temperature gradient: (**a**) cloud maps of the maximum principal stress distribution in conditions 3 and 7; (**b**) cloud diagram of stress changes in the retained track slab before and after slab-replacement operation.

Similarly, it is noteworthy that stress amplitudes in the track slabs are concentrated in relatively weak structural regions under the combined loading conditions of overall heating and positive temperature gradient, as shown in Figure 13a. Before the slab-replacement operation, the maximum stress amplitude is observed in the deteriorated slab slated for replacement, while, after the replacement, it shifts to the retained track slabs. Additionally, compared to the pre-replacement condition, the maximum tensile stress in the slabs adjacent to the new slab increases by 0.058 MPa, and the maximum compressive stress rises by 0.002 MPa. These slabs experience greater stress amplitudes, accelerating the accumulation of fatigue damage and potentially leading to premature structural failure.

To further quantify the changes in load transfer pathways in the retained track slab, stress differences at identical node positions before and after the slab-replacement operation were analyzed. As shown in Figure 13b, significant stress redistribution occurs in the retained track slabs. Specifically, tensile stress regions become concentrated near the slab ends at the connections with the anchorage structures. Notably, these locations lack internal reinforcement, and the altered load transfer pathways result in deviations between the actual stress state and the design intent of the internal reinforcement layout. This misalignment exceeds the collaborative capacity of the reinforcement and concrete, further undermining the structural integrity and increasing the risk of brittle failure in the track slabs.

**Figure 14:**
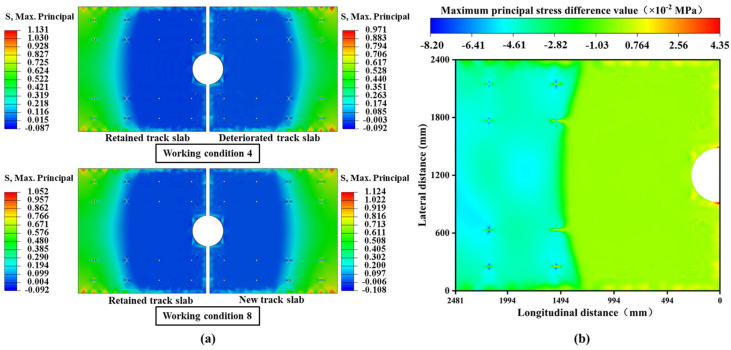
Coupling conditions of overall temperature rise and negative temperature gradient: (**a**) cloud maps of the maximum principal stress distribution in conditions 4 and 8; (**b**) cloud diagram of stress changes in retained track slab before and after slab-replacement operation.

Under the coupled loading conditions of overall heating and a negative temperature gradient, the maximum stress amplitudes before and after slab-replacement operations occur in structurally sound components, with a slight reduction in stress amplitudes observed in the retained track slabs. After the replacement, the peak tensile and compressive stresses are located in the new track slab, as illustrated in Figure 14a. The peak tensile and compressive stresses in the new slab exceed those in the remaining old slabs by 0.072 MPa and 0.016 MPa, respectively.

To further quantify changes in load transfer pathways in the retained track slabs, stress differences at identical node positions before and after the slab-replacement operation were analyzed. As shown in Figure 14b, significant stress redistribution occurs in the retained track slab following the replacement. Notably, tensile stress increases are observed at multiple locations, particularly at slab edges and ends, which lack reinforcement. Under this loading condition, the unreinforced regions of the retained track slab exhibit several areas with increased tensile stress, which may lead to the formation of through-thickness cracks at the edges. These cracks pose a risk to the structure’s durability and safety over time.

Overall, after structural replacement, the strength and stiffness of the system improve, transforming the longitudinal transition from “moderate strength–low strength–moderate strength” to a new configuration of “moderate strength–high strength–moderate strength.” Additionally, the elastic modulus of the contact materials at the connection points increases by 101.93% due to material replacement. This change creates a new transition zone that adversely affects load transfer. The stiffness difference between the reinforced and non-reinforced zones leads to stress concentration, which reduces the relative strength of surrounding areas and forms new weak points. Under various loading conditions, stress concentration effects emerge near bolt holes and the boss-shaped limiting device, potentially causing cracks or other types of damage.

Moreover, changes in local strength and connection elasticity disrupt the original load transfer pathways, resulting in a bypass effect. This effect involves the load bypassing the reinforced zones with higher strength and shifting toward adjacent weaker areas. Analysis reveals that tensile stresses are redistributed toward the edges and unreinforced regions under multiple loading conditions, deviating from the original design intent. This localized reinforcement approach can induce secondary damage, introducing new vulnerabilities to the structure. These issues warrant focused attention following the completion of slab-replacement operation to ensure structural integrity and durability.

## 6. Conclusions

To address the impact of strength and material differences on the structural behavior of critical components in CRTS I slab ballastless track before and after slab-replacement operations, numerical simulation methods were employed in conjunction with quantitative results. The effects of these differences on stress distribution and load transfer pathways were revealed. The main conclusions are summarized as follows:

1. Before the slab-replacement operation, the estimated concrete strength of the deteriorated track slab was 42.4 MPa, which is 10.74% lower than that of the adjacent retained track slab. After the slab-replacement operation, the estimated concrete strength of the new track slab was 59.5 MPa, 25.26% higher than that of the adjacent retained track slab. Regardless of the slab-replacement operation, the region exhibited uneven strength, which contributes to stress concentration issues.
2. Before the slab-replacement operation, the average elastic modulus of the old filling resin was 5.19 kN/mm, which is 35.13% below the minimum allowable design value. After the slab-replacement operation, the average elastic modulus of the new filling resin was 10.48 kN/mm, 31.00% above the minimum allowable design value. Significant differences in the filling resin materials on both sides of the limit devices result in abrupt changes in boundary conditions.
3. The replacement of track slabs targets regions with severe longitudinal damage, which is beneficial for train operation safety. However, localized reinforcement may lead to insufficient strength in surrounding areas, forming new weak points. Adjacent regions with lower stiffness may bear greater stress, potentially causing cracks or other types of damage. Meanwhile, the original load transfer path of the retained track slab is disrupted due to the replacement intervention, causing the load to bypass the newly reinforced high-strength region and shift toward adjacent weaker zones. Under multiple service conditions, this leads to tensile stress redistribution toward slab edges and unreinforced locations, which may in turn induce secondary deterioration. Accordingly, it is recommended that railway maintenance departments prioritize the routine inspection and monitoring of the critical regions following slab-replacement operations, particularly the interface between the ends of the retained slabs and the limit device, as well as the area near the embedded sleeves in the central section of the retained slabs. These areas should receive increased attention, especially during periods of prolonged high temperatures in summer and sustained low temperatures in winter, when more frequent safety inspections are advised to mitigate potential risks.

## Figures and Tables

**Table 1:** Model parameters of CRTS I slab ballastless track.

Components	Modulus of Elasticity/MPa	Poisson’s Ratio	Mass Density/kg·m^−3^	Coefficient of Linear Expansion/°C^−1^
Retained track slab	3.35 × 10^4^	0.2	2500	1.0 × 10^−5^
New track slab	3.6 × 10^4^	0.2	2500	1.0 × 10^−5^
Deteriorated track slab	3.25 × 10^4^	0.2	2500	1.0 × 10^−5^
CA mortar layer	300	0.2	2000	1.8 × 10^−5^
New filling resin	26.2	0.1	1200	2.0 × 10^−5^
Old filling resin	12.973	0.1	1200	2.0 × 10^−5^
Limit device	3.3 × 10^4^	0.2	1200	1.0 × 10^−5^
Base slab	3.3 × 10^4^	0.2	1200	1.0 × 10^−5^

**Table 2:** Model temperature load condition setting.

	Temperature Load		Working Condition
Before the slab-replacement operation	Lower the temperature by 30 °C	90 °C/m	Working condition 1
		−45 °C/m	Working condition 2
	Raise the temperature by 30 °C	90 °C/m	Working condition 3
		−45 °C/m	Working condition 4
After the slab-replacement operation	Lower the temperature by 30 °C	90 °C/m	Working condition 5
		−45 °C/m	Working condition 6
	Raise the temperature by 30 °C	90 °C/m	Working condition 7
		−45 °C/m	Working condition 8

## Data Availability

The original contributions presented in this study are included in the article. Further inquiries can be directed to the corresponding author.
